# An Intelligent Attention-Based Transfer Learning Model for Accurate Differentiation of Bone Marrow Stains to Diagnose Hematological Disorder

**DOI:** 10.3390/life13102091

**Published:** 2023-10-20

**Authors:** Hani Alshahrani, Gunjan Sharma, Vatsala Anand, Sheifali Gupta, Adel Sulaiman, M. A. Elmagzoub, Mana Saleh Al Reshan, Asadullah Shaikh, Ahmad Taher Azar

**Affiliations:** 1 Department of Computer Science, College of Computer Science and Information Systems, Najran University, Najran 66462, Saudi Arabia; hmalshahrani@nu.edu.sa (H.A.); aaalsulaiman@nu.edu.sa (A.S.); 2 Chitkara University Institute of Engineering and Technology, Chitkara University, Rajpura 140401, India; sharma.gunjan@chitkara.edu.in (G.S.); vatsala.anand@chitkara.edu.in (V.A.); sheifali.gupta@chitkara.edu.in (S.G.); 3 Department of Network and Communication Engineering, College of Computer Science and Information Systems, Najran University, Najran 61441, Saudi Arabia; meabdullah@nu.edu.sa; 4 Department of Information Systems, College of Computer Science and Information Systems, Najran University, Najran 66462, Saudi Arabia; msalreshan@nu.edu.sa (M.S.A.R.); asshaikh@nu.edu.sa (A.S.); 5 College of Computer and Information Sciences, Prince Sultan University, Riyadh 11586, Saudi Arabia; 6 Automated Systems and Soft Computing Lab (ASSCL), Prince Sultan University, Riyadh 11586, Saudi Arabia

**Keywords:** bone marrow cell, classification, transfer learning, medical research, deep learning, attention mechanism

## Abstract

Bone marrow (BM) is an essential part of the hematopoietic system, which generates all of the body’s blood cells and maintains the body’s overall health and immune system. The classification of bone marrow cells is pivotal in both clinical and research settings because many hematological diseases, such as leukemia, myelodysplastic syndromes, and anemias, are diagnosed based on specific abnormalities in the number, type, or morphology of bone marrow cells. There is a requirement for developing a robust deep-learning algorithm to diagnose bone marrow cells to keep a close check on them. This study proposes a framework for categorizing bone marrow cells into seven classes. In the proposed framework, five transfer learning models—DenseNet121, EfficientNetB5, ResNet50, Xception, and MobileNetV2—are implemented into the bone marrow dataset to classify them into seven classes. The best-performing DenseNet121 model was fine-tuned by adding one batch-normalization layer, one dropout layer, and two dense layers. The proposed fine-tuned DenseNet121 model was optimized using several optimizers, such as AdaGrad, AdaDelta, Adamax, RMSprop, and SGD, along with different batch sizes of 16, 32, 64, and 128. The fine-tuned DenseNet121 model was integrated with an attention mechanism to improve its performance by allowing the model to focus on the most relevant features or regions of the image, which can be particularly beneficial in medical imaging, where certain regions might have critical diagnostic information. The proposed fine-tuned and integrated DenseNet121 achieved the highest accuracy, with a training success rate of 99.97% and a testing success rate of 97.01%. The key hyperparameters, such as batch size, number of epochs, and different optimizers, were all considered for optimizing these pre-trained models to select the best model. This study will help in medical research to effectively classify the BM cells to prevent diseases like leukemia.

## 1. Introduction

Around four percent of a mature human’s body weight is densely innervated, heavily vascularized tissue known as bone marrow (BM). It is a critical part of the hematopoietic system in the human body, producing blood cells and being essential to the immune system [1]. Red and yellow bone marrow are the two different varieties of bone marrow. The red marrow predominantly synthesizes blood cells, including leukocytes, platelets, and red blood cells (erythrocytes). The yellow marrow, seen in the interior chambers of long bones, mainly comprises fat cells and functions as a reserve for storing energy [2]. Within the marrow, specialized hematopoietic stem cells (HSCs) can transform into different types of blood cells. HSCs can generate myeloid and lymphoid cells, which are crucial in immune system functioning [3]. Without a healthy BM, the body would not be able to create these vital blood cells, resulting in several health issues, including anemia. The generation of blood cells takes place in the BM.

The importance of BM is seen in its crucial function in preserving an effective immune system with a healthy blood supply. White blood cells (WBCs) aid in the fight against infections and disorders, platelets are important in blood clotting, and red blood cells provide oxygen to tissues and organs [2]. The red blood cells (RBCs) in a person’s body guarantee that oxygen gets transferred throughout the body’s cells, while platelets aid in clotting the blood in the event of an injury [4]. Numerous hematological disorders, including anemias, myelodysplastic syndromes, and leukemia, are diagnosed on the basis of certain anomalies in the number, kind, or appearance of bone marrow cells. Early and accurate identification of these disorders can be facilitated by a detailed classification of these cells. Any variation in the blood’s leukocyte (WBC) count indicates the reason for concern. The body can suffer and develop illnesses if WBC levels are unusually high [5]. So, it is crucial to maintain a careful check on the WBC count to guard against any health problems in the future.

It has been a standard practice for more than a century to analyze and classify bone marrow cell specimens using optical microscopes to diagnose blood disorders. BM biopsy is performed to keep a check on the count of cells. During a BM smear procedure, a small sample of bone marrow is collected from the patient’s hip bone (usually the posterior iliac crest) or sometimes from the breastbone (sternum). The procedure is typically performed by a hematologist or an oncologist in a hospital or clinic setting [6,7]. Therefore, a quick, reliable, and objective approach to the morphology of cell diagnosis is urgently needed.

Hematologists now frequently employ computer-aided diagnostics and testing to support them in examining blood cell images. These instruments can employ computer-assisted microscopy techniques to produce an analysis that appears more precise and uniform [3]. Image processing technology improves the speed and precision of human procedures and saves time, money, and resources. It is a cutting-edge method that combines computer technology, artificial intelligence, digital picture processing, and blood smear-based analysis [8].

This work utilizes a transfer learning approach to identify and count various cells in a blood smear automatically. The suggested approach reduces inspection time, the impact of human variables, and the possibility of an error in counting because of wariness. This study utilizes fine-tuned pre-trained models to achieve the best accuracy model with specified hyper-parameters. Pre-trained models, like EfficientNetB5, MobileNetV2, DenseNet121, ResNet50, and the Xception model, were used to classify BM cells into seven categories. For the classification, a BM cell classification dataset has been used. The best-performance model was retrained by applying different optimizers and a modified value for the batch size, and then further integrating it with an attention mechanism. The following points highlight the critical contributions of the planned research.

- In this study, five TL models—EfficientNetB5, ResNet50, DenseNet121, Xception, and MobileNetV2—are fine-tuned by adding a batch-normalization layer, a dropout layer, and two dense layers for the BM cell classification task.
- The performance of five fine-tuned TL models was evaluated in terms of their accuracy and loss, out of which the DenseNet121 model performed best.
- The fine-tuned DenseNet121 model was optimized using several optimizers, such as AdaGrad, AdaDelta, Adamax, RMSprop, and SGD, along with different batch sizes of 16, 32, 64, and 128.
- Furthermore, the fine-tuned and optimized DenseNet121 model was integrated with an attention mechanism to improve its classification performance. By leveraging the attention mechanism’s ability to emphasize the important features and DenseNet121’s ability to concatenate feature maps iteratively, this fusion provides a potent combination for precise and comprehensible categorization.

## 2. Literature Review

Analyses of medical images have recently made substantial use of convolutional neural networks (CNN) and deep learning (DL). Naturally, given that it offers a solution to the requirement for big-sized training datasets, the use of transfer learning (TL) is also encouraged in this discipline [9]. To provide the most up-to-date information on this topic, Morid, Mohammad Amin, et al. [10] examined a few studies using various ImageNet-trained transfer learning models to interpret clinical images. Their investigation suggests that the employment of models relies on the kind of photos being analyzed. Faruk, Omar et al. [11] used four distinct transfer learning models to identify tuberculosis employing X-ray images: MobileNetV2, InceptionResNetV2, InceptionV3, and Xception. In their study, three MaxPooling2D layers, four Conv2D layers, a flattened layer, two dense layers, and the activation function of ReLU were all present in each model.

Regarding the accuracy (99.36%), InceptionResNetV2 performed better than the other models. The leukocyte classification approach employing segmentation and modified ResNet50-based categorization, as suggested in [12], offers test accuracy above 90%. For the categorization of the blood cells, the research by [13] developed a model that merged the deep convolutional generative adversarial network (DC-GAN) and the ResNet model using a transfer learning strategy on the ImageNet dataset. The outcomes reveal a 1.2% improvement in its accuracy on DC-GAN-enriched images and a 91.68% testing accuracy overall. This study [14] used a CNN-based method that used the VGG16 and InceptionV3 algorithms to categorize blood cell types using 17,902 digital images and 8 classifications. Although both techniques had 90% overall accuracy, there was a significant difference in the rate of true positives for different groups. Another research effort [15] suggested using a deep learning framework based on patches to quickly localize bone marrow and create ROIs to categorize 16 types of cells. The suggested model has an overall validation recall of over 90% and has been trained using 12,426 labelled cells. Test findings on a second batch of 3000 photos, which achieved an accuracy rate of over 98%, demonstrate that the predictive model does not overfit the training set. 

In specific research, white blood cells are located on microscopic views using image-segmentation techniques. In [16], RGB color space input photos were transformed into the hematoxylin eosin-diaminobenzidine (DAB) format. Then, a double filter with canny edge segmentation was applied to recover the individual lymphocytes. The seed of every part was eventually determined using a watershed method. The accuracy of this technique was over 90%. The research conducted by [17] employed SoftMax to categorize the acute form of leukemia into various subtypes along with normal states and suggested the construction of neural networks with deep layers utilizing CNN’s AlexNet model. The accuracy was 97.78% on the 330-piece test set.

Further, the authors in [18] employed ImageNet rather than AlexNet and found 33 photos from the “Acute Lymphoblastic Leukemia” database (ALL-IDB). The algorithm accurately recognized the lymphoblasts for about 94.1%. In [19], ALL classification was performed using a convolutional neural network ResNeXt50 with a squeeze-and-excitation module. At first, ImageNet was employed for pre-training of the network. An overall precision of 89.7% was attained.

The initial approach for ALL identification, which utilizes histopathological transferred learning, was presented by Genovese et al. [20]. Before being optimized on the ALL database, CNN underwent training on histopathology databases to identify the various lymphoblast tissues and their types, having an accuracy rating of 88.69%. A CNN was used by [21] for classifying different WBC types to detect ALL, and previously trained DL frameworks, like AlexNet, GoogleNet, and VGG-16, were contrasted against one another to determine the model that could classify the most accurately, having a 96.15% accuracy rate. A technique [22] for leukemia detection was developed utilizing the Apache Spark BigDL package and the CNN framework for GoogleNet deep transfer learning. A 96.42% accuracy rate was attained using microscopic images of the human corpuscle. Employing transfer learning frameworks, Ref. [23] claimed to have 100% accuracy in leukemia detection. Two models were used for their study. To detect automated bone marrow cytology, the You-Only-Look-Once (YOLO) framework was put out by [24]. From a digitized full-slide picture of bone marrow, the framework recognizes and classifies every bone marrow cell by automatically recognizing the areas appropriate for cytology. In terms of area detection (the ROC curves was around 0.97 and AUC was 0.99, which indicates the accuracy levels), cell identification and classification were given by a mean average precision of 0.75 and an average F1-score of 0.78, respectively, suggesting that the system demonstrated outstanding accuracy. A CNN was employed by the contributors of the article [25] to categorize different leukemia kinds. Additionally, the dataset was diversified by using data augmentation. Consequently, 231 test samples were used to categorize all leukemia variants with a rate of 81% accuracy. Cross-validation was further used in each trial. Researchers from [26] suggested using the Siamese network to categorize WBC. Implementing the Siamese network framework, basophils and eosinophil cells were classified with an estimated accuracy of about 89.66%. The research by [27] suggested a categorization system for identifying WBC nuclei and nucleus characteristics. They employed an amalgamated classifier based on SVM along with a network of neural networks. They were almost 100% successful in identifying WBC types like lymphocytes and basophils where the cytoplasm was either absent or sparsely present. To categorize BM cell structure, Ref. [28] suggested the ResNeXt model. Using the same topological structure, ResNeXt duplicates a structural element that integrates many transformations. A comprehensive investigation of the various approaches and difficulties encountered in classifying WBC classes from a microscopic view of blood smear images was published by [29]. According to authors of [30], the main obstacle to leukocyte categorization is the precise identification of various images. They introduced a transfer learning strategy utilizing ResNet50 and an SVM-based classifier for WBC identification and classification, which entails data enhancement to increase precision.

In this study, a bone marrow (BM) cell categorization technique has been proposed to classify BW cells into seven categories using a dataset that contains images of BM biopsies of different patients. This enabled us to apply the information achieved from one activity to other similar ones. Any model based on deep learning may be used for an image classification approach employing transfer-based learning as a means of feature extraction. 

## 3. Proposed Methodology

**Figure 1:**
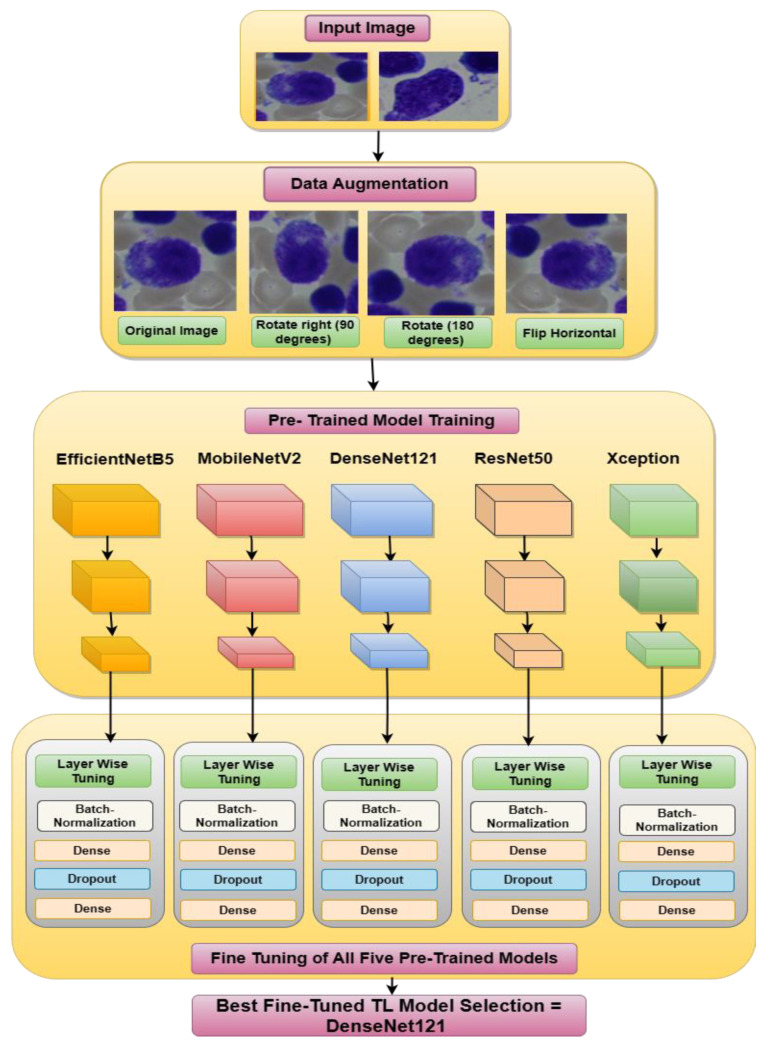
Proposed methodology for best TL model selection.

**Figure 2:**
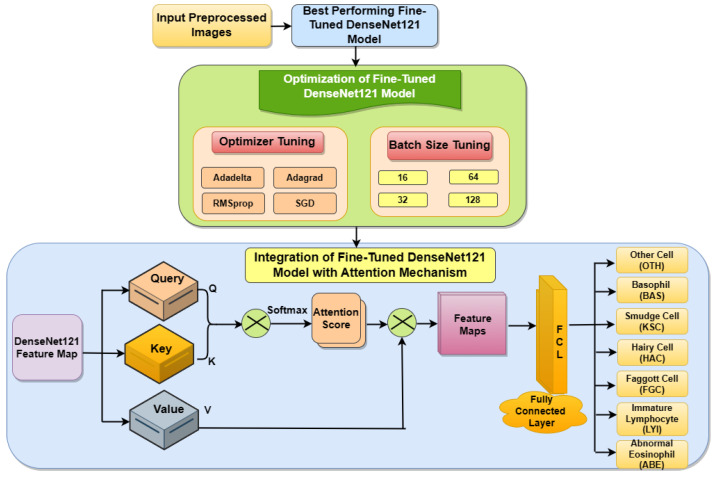
Proposed fine-tuned DenseNet121 model with integration of attention mechanism.

The proposed methodology is divided into two sections, as shown in Figure 1 and Figure 2. Figure 1 shows the data augmentation, the fine-tuning of five TL models, and selection of the best fine-tuned TL model. Figure 2 shows the optimization and integration of the best-performing DenseNet121 model with an attention mechanism. This methodology aims to develop a transfer learning classification workflow that is both precise and effective [20]. It can separate the input BM cell images into seven classes: abnormal eosinophils, faggot cells, immature lymphocytes, hairy cells, basophils, smudge cells, and other cells [31]. As can be seen in Figure 1, preprocessing steps are performed on the images before they are fed to the network in order to eliminate noise and data imbalances, allowing the model to be trained for the classification task more successfully. To balance the classes where the images are flipped both vertically and horizontally with distinct angles, a data augmentation technique is used. Furthermore, augmented images are fed to five TL models: Xception, EfficientNetB5, DenseNet121, ResNet50 and MobileNetV2, which are fine-tuned by adding a batch normalization layer, a dropout layer, and two dense layers. Subsequently, the images are ready for feature extraction and training. The BM cell classification dataset was used for training and testing of all these models, and accuracy and loss were used to assess each model’s performance individually. From the analysis of the results, it was noticed that the DenseNet121 performance outperformed the other models’ performances. Ultimately, DenseNet121 is the best fine-tuned TL model selection, as determined by the findings.

Figure 2 shows the proposed fine-tuned DenseNet121 model with the integration of an attention mechanism. Here, the pre-processed images are passed to the best-performing fine-tuned DenseNet121 model.

In the next step, this model is optimized with four different optimizers. A variety of optimizers are used in this optimization step, including AdaGrad, which modifies learning rates based on previous gradients; SGD, which iteratively updates weights using data subsets; RMSprop, which modifies AdagGrad for non-static objectives; and AdaDelta, which corrects AdaGrad’s diminishing learning rate. Different batch sizes (16, 32, 64, and 128) were explored in conjunction with these optimizers. After this, the integration of the DenseNet121 model takes place by adding an attention mechanism [32] to improve performance by allowing the model to focus on the most relevant features or regions of the image. The attention mechanism recapitulates the context-based data in the input sequence with variable length. Self-attention implements attention to a single context of data as compared to multiple contexts, therefore allowing direct long-distance interdependency. The proposed fine-tuned DenseNet121 model with self-attention, as illustrated in Figure 2, divides the DenseNet121 input feature map into three parts: Query, Key, and Value of the same shape as (Bsize × LQuery × Dmodel). These are the mean batch size, query length, and model dimension, respectively. The attention score was obtained by first convolution of the Query and Key, and then passing through the soft-max layer. The result of the attention process is the attention score, which is further convoluted with the Value. The feature map obtained through convolution process is passed to a fully connected layer. The final output of self-attention is used for the classification of BM cells into seven classes through the fully connected layer.

### 3.1. Dataset

**Figure 3:**
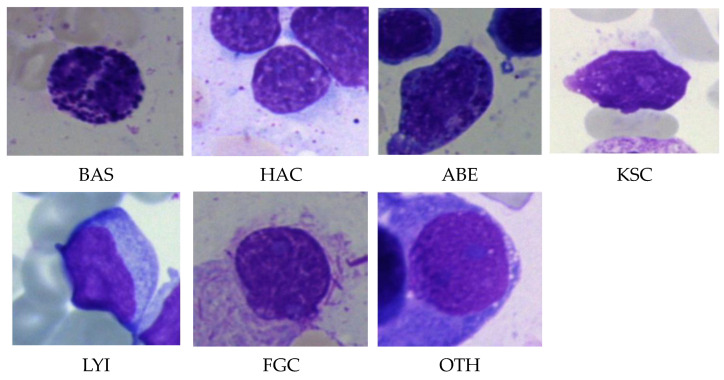
Sample images with a 40-fold magnification of 7 classes from the dataset [28].

This work used the bone marrow cell classification dataset from Kaggle [28]. Applying May–Grunewald–Giemsa staining, about 170,000 labeled images of BM stains from 945 individuals were included in the collection. The images were captured using a light microscope with a 40-fold magnification and oil immersion. According to each image’s hematological condition or feature, there are 21 different categories for the BM, out of which, 7 classes were chosen for this study. These categories, along with their abbreviations, are listed in Table 1. Figure 3 shows sample images of these classes.

### 3.2. Data Augmentation for Balancing

Data balancing is necessary to solve the problem of class imbalance in datasets. Class imbalance occurs when there is a significant skew in the distribution of classes within a dataset, with one or more classes having disproportionately fewer samples than the others [11]. It affects the performance of the image classification model. A class disparity may result in biased model performance [33]. Because they are exposed to more examples of that class during training, machine learning models often have higher accuracy in predicting the majority class. As a result, the minority class may be neglected or incorrectly categorized, resulting in subpar performance. Imbalanced datasets can produce decision boundaries skewed towards the dominant class. This indicates that even if the model achieves high accuracy, it can have trouble generalizing successfully to new data, especially when the minority class is of major concern. The model learns more representative decision limits and improves generalization when the dataset is balanced [28]. 

**Figure 4:**
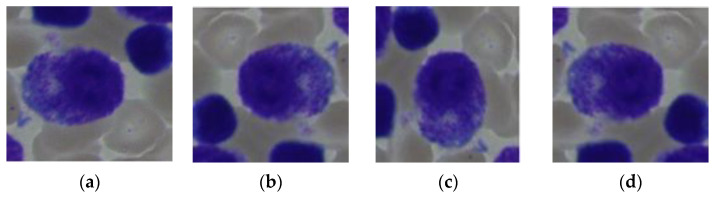
Data-augmented sample images with a 40-fold magnification: (**a**) original image; (**b**) 180° rotated image; (**c**) 90° rotated image; (**d**) flipped vertical image.

Various methods, such as oversampling, under-sampling, data augmentation, and class weight adjustment, can be applied for data balancing [34]. For this study, a data augmentation technique was used to balance the dataset. Increasing the amount of a training dataset artificially using modified versions of existing data samples is known as data augmentation. It is a frequent approach in machine learning and deep learning [33]. The procedure entails altering the original data in various ways while keeping the information that makes up those data intact. Images can be randomly cropped, flipped, rotated, scaled, sheared, and given noise or blur using data augmentation techniques. These modifications can produce new variants of the original photos, such as various orientations, viewpoints, or lighting conditions. Figure 4 shows the data-augmented image samples. In Table 1, it can be seen that the classes had significantly fewer images. So with the help of data augmentation, the number of images were increased in those classes. Table 2 describes the numbers of increased images in the classes.

### 3.3. Fine-Tuned TL Models for the Detection and Classification of Bone Marrow 

The need for more data is among the major issues facing the area of medical research. However, transfer learning may be used to solve this issue. By transferring the information from a pre-trained model to a new model, the transfer learning (TL) approach reduces the requirement for a big dataset [35]. Here, five transfer learning models were applied to the BM cells images to classify them into seven classes. The TL models used here were EfficientNetB5, MobilenetV2, DenseNet121, ResNet50, and Xception. 

#### 3.3.1. Fine-Tuned EfficientNetB5

**Figure 5:**
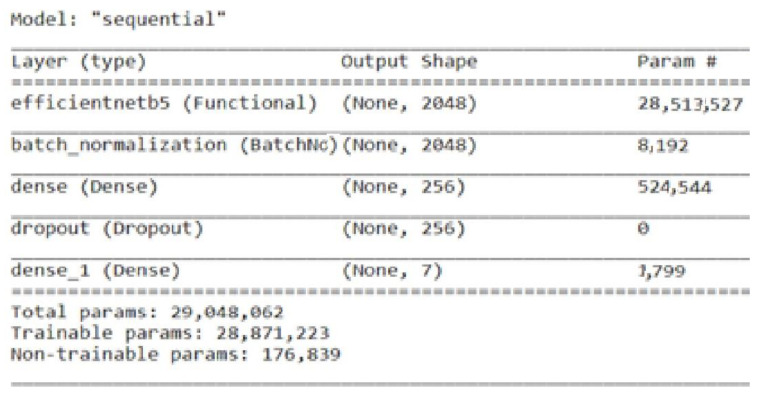
Parameters of fine-tuned EfficientNetB5 model.

A class of convolutional neural networks called EfficientNet was created to deliver cutting-edge performance with effective resource management. One particular version of the EfficientNet model is EfficientNetB5. The basic architecture’s scaling factor is denoted by the “B5” in EfficientNetB5. The scaling factor governs the network’s depth, breadth, and resolution. It has proven effective in utilizing EfficientNetB5 for various computer vision applications, such as picture segmentation, object identification, and categorization of images [36]. The EfficientNetB5 is fine-tuned by adding a batch normalization layer with a size of 2048, one dense and dropout layer with a size of 256, and one dense output layer for seven classes. Figure 5 represents the total parameters, including the trainable and non-trainable parameters used here in the fine-tuned EfficientNetB5 to classify the BM dataset.

#### 3.3.2. Fine-Tuned MobilenetV2

**Figure 6:**
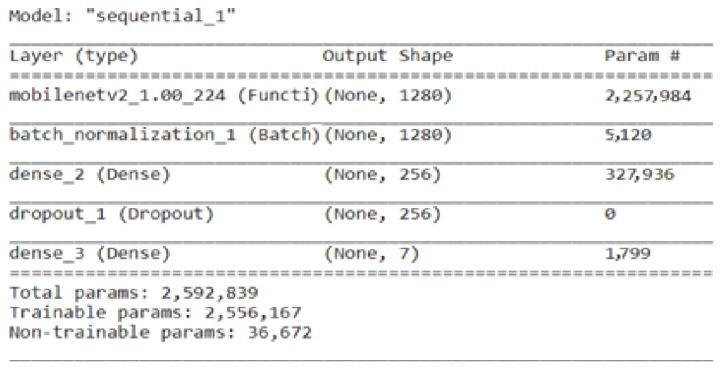
Parameters of fine-tuned MobileNetV2.

The basic concept of MobileNetV2 is to lower the computation complexity while preserving performance by combining depth-wise separable convolutions with linear bottlenecks. Convolution is divided into two independent layers by MobileNetV2. Each channel is individually subjected to a depth-wise convolution in the first layer, known as the depth-wise convolution [37]. The outputs of the depth-wise convolution are combined in the second layer, referred to as the point-wise convolution, using a 1 × 1 convolution. By factorizing the convolution procedure, this division minimizes the number of calculations. [20]. The MobileNetV2 pre-trained model is further fine-tuned by adding a batch normalization layer, a dense layer with a size of 256, along with a dropout layer, and a last dense layer with a size of 7. Figure 6 represents the total parameters, including the trainable and non-trainable parameters used here in the fine-tuned MobileNetV2 for classifying the BM dataset into seven classes.

#### 3.3.3. Fine-Tuned DenseNet121

**Figure 7:**
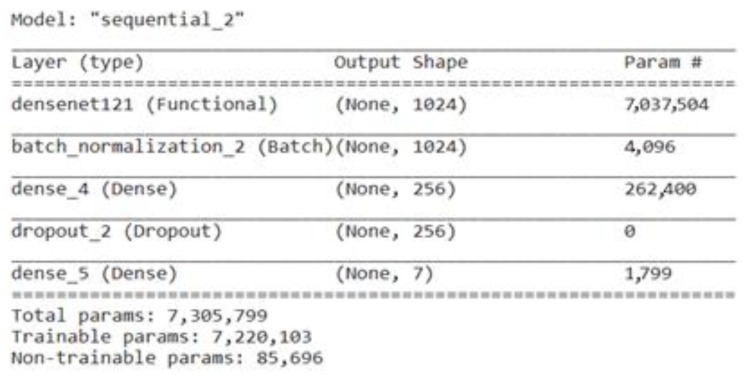
Parameters of fine-tuned DenseNet121 model.

DenseNet refers to a “Densely Connected Convolutional Network”, which is renowned for its superior performance in image classification tasks. By creating dense connections between layers, DenseNet aims to solve the issue of vanishing gradients and promote feature reuse [38]. Each layer in a DenseNet is feed-forward directly linked to every other layer, creating dense connections (also known as skip connections) [30]. DenseNet-121 outperformed other well-known designs while keeping a manageably low number of parameters in various image classification evaluations. This DenseNet121 model was fine-tuned by adding a batch-normalization layer, a dense layer with a size of 256, a dropout layer, and last dense layer with a size of 7. Figure 7 represents the total parameters, including the trainable and non-trainable parameters used here in the fine-tuned DenseNet121.

#### 3.3.4. Fine-Tuned ResNet50

**Figure 8:**
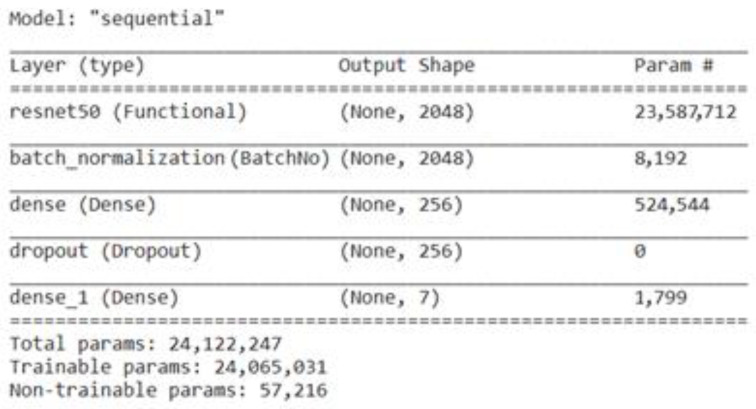
Parameter of fine-tuned ResNet50 model.

The inclusion of residual connections, also known as skip connections, is the main innovation of ResNet-50 and the ResNet family. These linkages make bypassing some layers and immediately propagating input from one layer to a deeper layer possible. As a result, the network is better equipped to deal with the vanishing gradient issue and lessen the degradation problem that frequently occurs with deeper networks. The residual block, which has two or three convolutional layers and identity shortcut connections, is the fundamental component of ResNet-50 [39]. ResNet50 is fine-tuned by adding a batch-normalization layer, a dense layer with a size of 256, a dropout layer, and a last dense layer with a size of 7. Figure 8 represents the total parameters, including the trainable and non-trainable parameters used here in the fine-tuned ResNet50 to classify the BM dataset into seven classes.

#### 3.3.5. Fine-Tuned Xception

**Figure 9:**
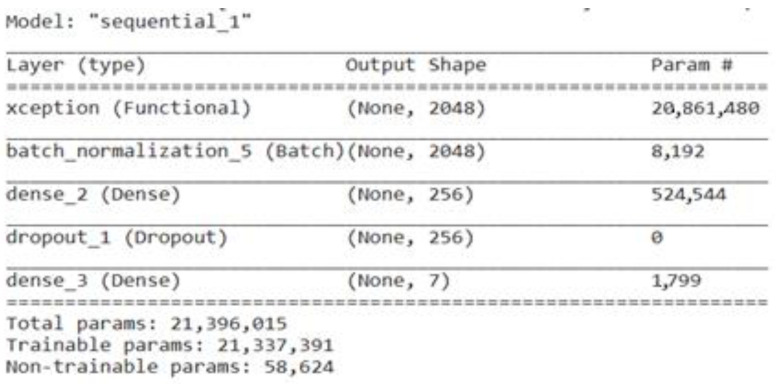
Parameters of fine-tuned Xception model.

François Chollet, the Keras deep learning package developer, proposed the CNN architecture known as Xception. To attain a high degree of performance and efficiency, the central concept underlying Xception is to make use of depth-wise separable convolution, which was initially presented in the MobileNet architecture [39]. The spatial and channel-wise convolutions are separated by depth-wise separable convolutions, which reduce computing complexity while maintaining crucial data. It provides a strong balance between accuracy and computing efficiency, making it appropriate for deployment on devices with limited resources or in situations where real-time processing is required. The Xception model is fine-tuned by adding a batch-normalization layer, a dense layer with a size of 256, a dropout layer, and a last dense layer with a size of 7. Figure 9 represents the total parameters, including the trainable and non-trainable parameters of the fine-tuned Xception model.

### 3.4. Selection of Best Fine-Tuned TL Model

The performances of all five fine-tuned TL models were analyzed in terms of accuracy and loss curves, from which it was concluded that the DenseNet121 model outperformed the other TL models in terms of both accuracy and loss. A graphical analysis of all TL models is given in Section 4.1. 

### 3.5. Different Optimizers Employed for Best Fine-Tuned TL Model

The parameters of the best DenseNet121 model are modified during the training process using an optimization technique. By modifying the model’s parameters based on the gradients of the loss function concerning those parameters, an optimizer seeks to minimize a defined loss or error function [12]. Here, different optimizers were used to optimize the performance of the best TL model. In this investigation, the five different optimizers used were Adamax, AdaGrad, SGD, RMSprop, and AdaDelta. A comprehensive examination of model performance with different optimizers was then achieved through comparison. 

The Adamax optimizer is a variation of the Adam optimizer, which excels at solving problems with huge parameter spaces and sparse gradients [36]. An optimization approach called Adaptive Gradient (AdaGrad) adjusts the learning rate of each parameter based on previous gradients [37]. The fundamental idea underlying AdaGrad is to provide each parameter with a unique learning rate dependent on the size of its previous gradient. SGD is a well-known optimization approach that modifies the model’s parameters in tiny increments proportionate to the loss function’s negative gradient [38]. It is a straightforward and often employed optimizer, although it can converge slowly and is sensitive to the learning rate. The optimization approach Root Mean Square Propagation (RMSprop) overcomes AdaGrad’s drawbacks of aggressive and monotonically declining learning rates [37]. It is intended to enhance optimization processes’ convergence and stability, particularly for deep learning models. AdaDelta is a deep learning model training optimization technique. It is an AdaGrad algorithm extension that tackles some of its shortcomings. AdaGrad adjusts the learning rate depending on the sum of squared gradients for each parameter, which can result in a declining learning rate and unstable convergence [39].

### 3.6. Integration of DenseNet121 Feature Map with Attention Mechanism

This section describes the integration of the fine-tuned DenseNet121 model with an attention mechanism. The concept of self-attention involves the application of attention mechanisms to a singular context, as opposed to multiple contexts [32]. This approach facilitates the establishment of direct interdependencies over great distances. Attention mechanisms are frequently employed in computer vision to supplement the CNN model. The analysis centers on a certain characteristic that holds significance in the process of classification. In the self-attention mechanism, the input feature map is divided into three parts: Query (Q), Key (K), and Value (V), which have the same dimensions of batch size, query length, and model dimension. Initially, the attention score is obtained by convolution of the Query and Key. The result of the attention process is the attention score, which is further convoluted with the Value, as shown in Equation (1).

(1) $$attention\left(Q,K,V\right)=\sum_{i}similarity\left(Q,K_{i}\right)*V$$

where K**_i_** is the key value for the i^th^ iteration. The attention mechanism measures the similarity between the Query Q and each Key value K**_i_**. This similarity returns a weight for each Key value. Finally, it produces an output that is the weighted combination of all the values in our database. Here, the DenseNet121 feature map was divided into three parts, Query, Key, and Value, to perform an attention mechanism to improve its performance. 

## 4. Results and Discussion

The BM cell classification dataset was used to train and simulate five pre-trained and fine-tuned models. These models were Xception, ResNet50, DenseNet121, MobileNetv2, and EfficientNetB5. 

### 4.1. Graphical Representation of Fine-Tuned Transfer Learning Models Performance

Figure 4 illustrates the graphical results of all five fine-tuned pre-trained models. At first, by utilizing the Adamax optimizer, these TL models were trained for 25 iterations with a Batch size of 40. Loss vs. Epoch (training and testing) and Accuracy vs. Epoch (training and testing) graphs are shown. In the graphs, a blue dot displayed the best epoch for which the model’s performance was optimum. Here, the green color represents the testing values, and the red color signifies the training values. 

**Figure 10:**
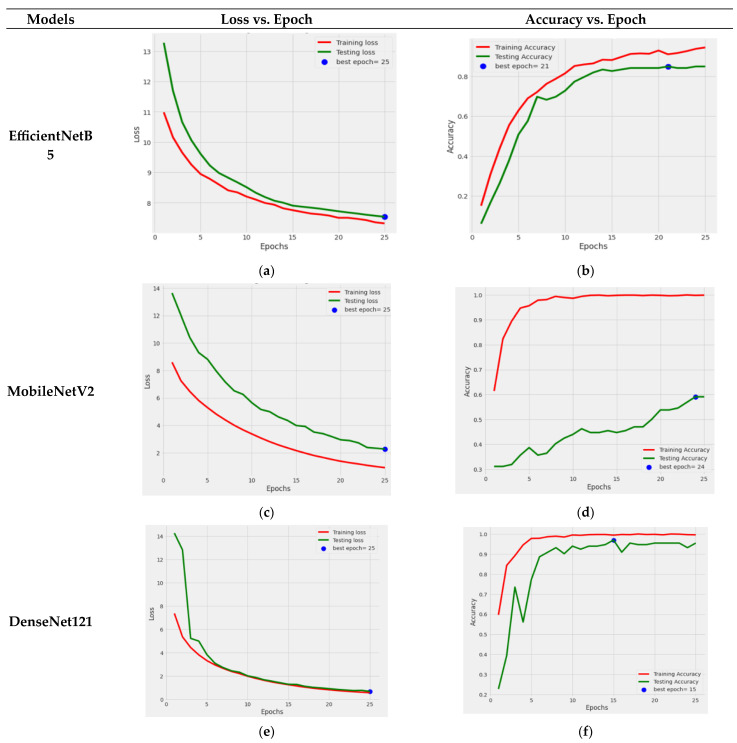

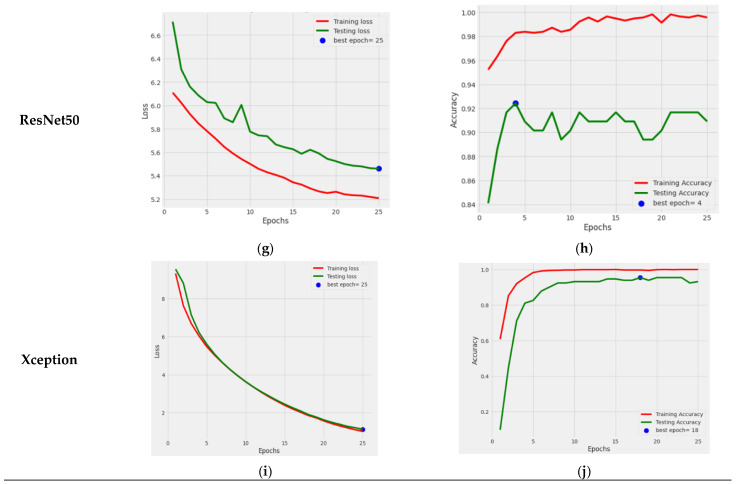
Graphical representation of training and testing loss of different fine-tuned TL models: (**a**) EfficientNetB5; (**c**) MobileNetV2; (**e**) Densenet121; (**g**) ResNet50; (**i**) Xception. Training and testing accuracy of different TL models: (**b**) EfficientNetB5; (**d**) MobileNetV2; (**f**) DenseNet121; (**h**) ResNet50; (**j**) Xception.

Figure 10a,c,e,g,h displays the graphs of the training and testing loss of the fine-tuned EfficientNetB5, MobileNetV2, DenseNet121, ResNet50, and Xception pre-trained models, respectively. It can be seen in the graph that the training loss and testing loss consequently decreased for all pre-trained models. At the same time, Figure 10b,d,f,h,i shows a graph of the training and testing accuracy of the models. Figure 10b shows that the training and testing accuracy of the EfficientNet model consistently rose. The model had its highest value on the 23rd epoch. Figure 10d shows that the testing accuracy of the MobileNetV2 model stayed the same at 59%. Figure 10f shows that the DenseNet121 model’s training accuracy reached nearly 99%, and its testing accuracy reached 95.45%. The best performance epoch was 20 for this model. ResNet50 model’s accuracy can be seen in Figure 10h, from which it can be seen that the testing performance of this model reached a value of 90%. At last, Figure 10j represents the Xception TL model’ accuracy, which consistently rose and reached 93.18%.

From the graphs, it can be seen that the performance of the DenseNet121 model was the best among the other TL models in terms of loss and accuracy.

### 4.2. Graphical Performance Representation of Diverse Optimizers with Best Transfer Learning Model 

**Figure 11:**
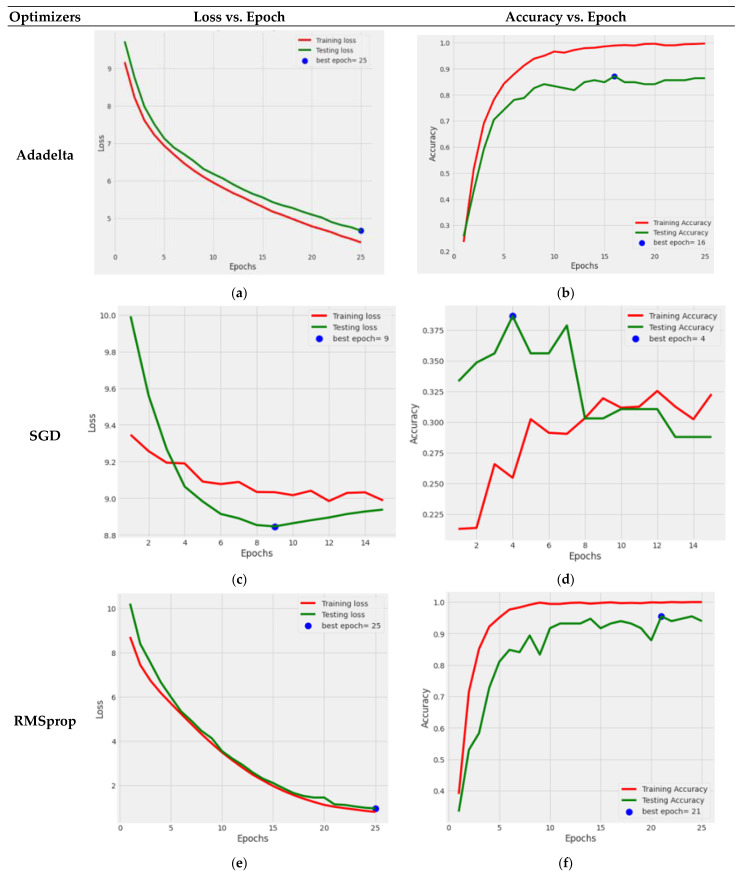

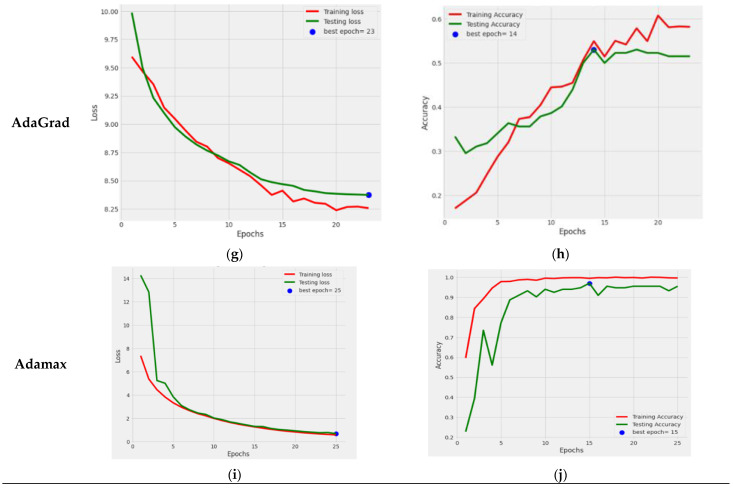
Graphical representation of training and testing loss of fine-tuned DenseNet121 with different optimizers: (**a**) AdaDelta; (**c**) SGD; (**e**) RMSprop; (**g**) AdaGrad; (**i**) Adamax. Training and testing accuracy of different optimizers: (**b**) AdaDelta; (**d**) SGD; (**f**) RMSprop; (**h**) AdaGrad; (**j**) Adamax.

Figure 11 depicts the graphical depiction of the DenseNet121 Model with five optimizers: AdaDelta, SGD, RMSprop, AdaGrad, and Adamax.

The blue dots in the graphs reflect the best epoch count for which the model’s performance was optimal. The green color reflects the testing loss and accuracy, whereas the red color indicates the training loss and accuracy. Figure 11a,b gives the loss and accuracy performance graph with the use of an AdaDelta optimizer; from this Figure, it can be concluded that both the training and testing losses steadily decreased, while the training accuracy improved smoothly, and the testing accuracy increased but with tiny ups and downs. The performance of the SGD optimizer can be seen in Figure 11c,d, where the testing loss decreased until the ninth epoch, and then it increased. In contrast, the value of the training loss decreased with every passing epoch. The accuracy graph may be more satisfactory since the training and testing accuracy numbers vary significantly. Because of this lack of development, the model training was discontinued after the 15th epoch. Figure 11e,f clearly shows that the model performed well in categorizing the BM cells into seven classes using the RMSprop optimizer. The losses diminished one by one, the training accuracy grew to a final value of 99.97, and the testing accuracy results reached 93.93. The AdaGrad optimizer graph shown in Figure 11g,h indicates that the testing and training loss values steadily reduce. However, significant variations in training and testing accuracy were observed. The training was terminated at the 24th epoch since there was no improvement in the values. Figure 11i,j shows that with the implication of the Adamax optimizer, DenseNet121 performed exceptionally well, with a testing accuracy of 95.45% and a training accuracy of nearly 99%.

So, from Figure 11, it is concluded that the fine-tuned DenseNet121 performed very effectively with the Adamax optimizer.

### 4.3. Graphical Performance Representation of Diverse Batch Size with Best Fine-Tuned Transfer Learning Model

**Figure 12:**
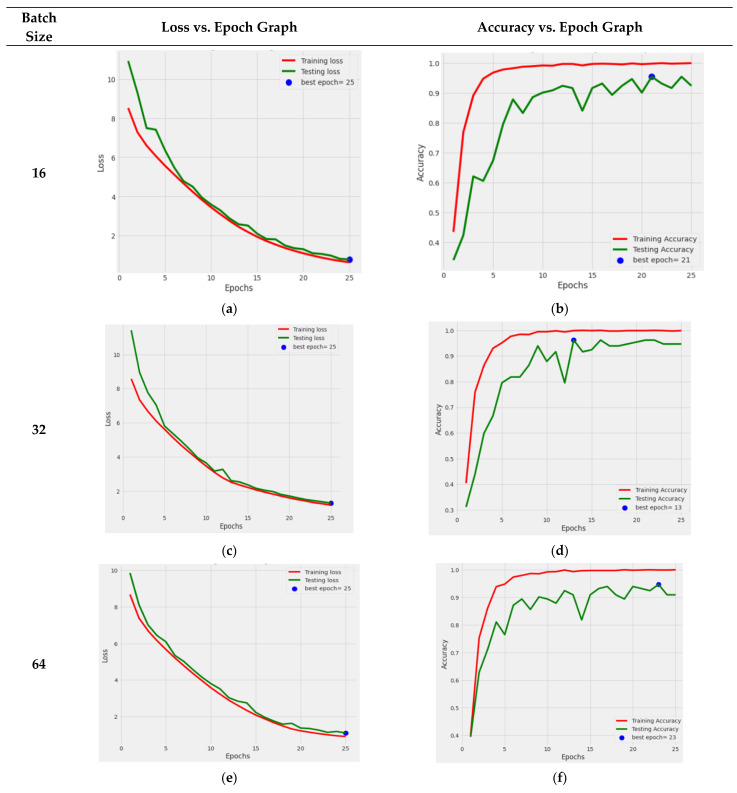

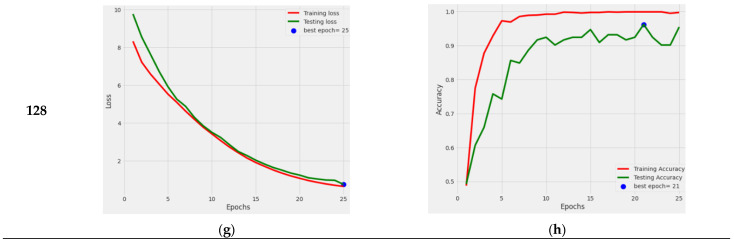
Graphical representation of training and testing loss of DenseNet121 model with different batch sizes: (**a**) batch size 16; (**c**) batch size 32; (**e**) batch size 64; (**g**) batch size 128. Training and testing accuracy of different batch sizes: (**b**) batch size 16; (**d**) batch size 32; (**f**) batch size 64; (**h**) batch size 128.

It can be deduced from Figure 11 that the DenseNet121 TL model outperformed the others when the Adamax optimizer was used. The Dense-Net121 TL model was retrained using the Adamax optimizer and various batch sizes of 16, 32, 64, and 128 to provide more precise results. The graphical depiction of DenseNet121’s performance is shown in Figure 12.

The performance graph of DenseNet121 with the Adamax optimizer and a batch size of 16 is shown in Figure 12a,b. The training and testing losses both steadily decreased, and the training accuracy became better with each passing epoch, while the testing accuracy value increased, with some ups and downs. The loss and accuracy graph of batch size 32 is shown in Figure 12c,d. From this, it can be seen that both losses continuously reduced, while the testing accuracy significantly fluctuated. The ideal epoch was 13, where the testing accuracy was at its greatest before slightly declining in value. The loss and accuracy graph for the 64 batch size is shown in Figure 12e,f. With little variations in the value, it can be observed that test accuracy rose. The ideal period when the testing accuracy was the most remarkable, was 25. The outcomes shown in Figure 12g,h are for a batch size of 128. From this graph, it can be seen that the losses steadily declined, while the testing accuracy value steadily rose. Here, when the testing accuracy was at its maximum, the optimal epoch count was 21.

### 4.4. Results of DenseNet121 Model Integrated with Attention Mechanism

**Figure 13:**
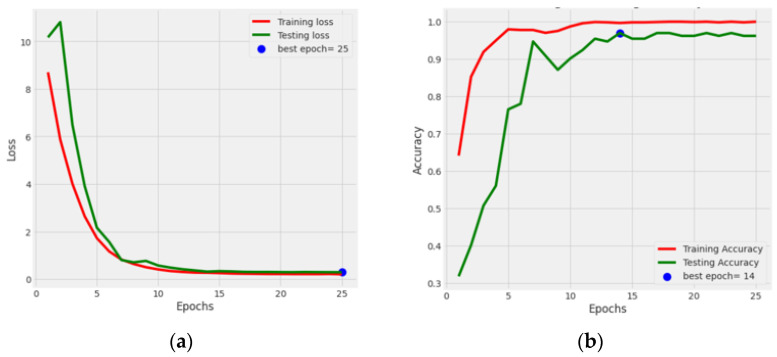
Graphical representation of integrated DenseNet121 model: (**a**) training and testing loss; (**b**) training and testing accuracy.

This section showcases the results of the fine-tuned DenseNet121 integrated with an attention mechanism optimized with a batch size of 32, optimizer Adamax, and 25 epochs. Figure 13a displays the loss of the training and testing, and Figure 13b displays the training and testing accuracy. In Figure 13a, it can be noticed that the training and testing loss value diminished. The testing loss reached a minimum value of 0.09. In Figure 13b, it can be seen that the model performance increased to 97.01%, and the training accuracy reached 99.80% after the implementation of the attention mechanism. From Figure 13, it can be concluded that the optimized and fine-tuned DenseNet121 model integrated with the attention mechanism gave a better performance than the standard DenseNet121 model. From these new results, it can be concluded that by adding an attention mechanism to the fine-tuned DenseNet121 model, the model’s performance increased from 95.45% to 97.01%.

### 4.5. Visualization of Classification and Misclassification Results

**Figure 14:**
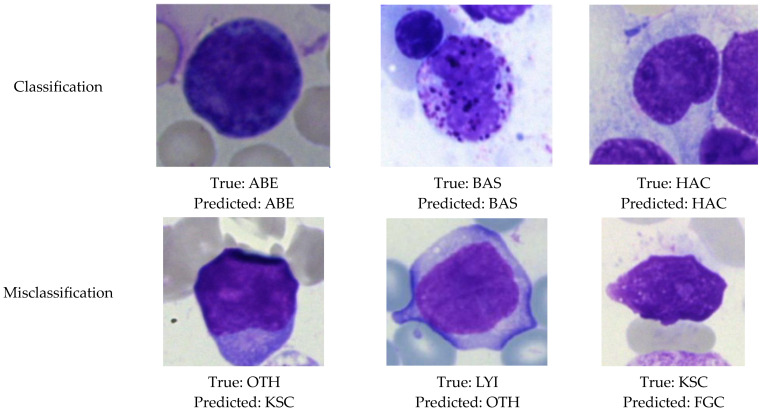
Results of classification and misclassification.

In this section, the results of the classification and misclassification of the BM cells re shown. For the classification of BM cells, a CNN model based on the DenseNet121 transfer learning model was proposed to categorize the BM cells into seven classes. Figure 14 displays the result of the classification and misclassification of images of multiple classes.

### 4.6. State of Art Comparison

In this section, the performance of the proposed model is contrasted with the findings of some other investigators in categorizing BM cells. The integrated fine-tuned DenseNet121 model achieved the highest accuracy of 97.01%, surpassing all other models, including ResNet50, VGG16 with InceptionV2, Siamese Network for Few Shot, and ResNeXt. The accuracy of the DenseNet121 model (97.01%) was notably higher than that of the other techniques, showcasing its effectiveness in accurately classifying images. The closest competitor to the DenseNet121 model’s accuracy is the DC-GAN and ResNet technique, with an accuracy of 91.68%. However, the DenseNet121 model still outperformed it by a significant margin.

The Siamese Network achieved an accuracy of 89.66%, which is slightly lower than the DenseNet121 model’s accuracy. The ResNeXt model achieved an accuracy of 94.8%, which is lower than the DenseNet121 model’s accuracy.

In summary, the proposed integrated fine-tuned DenseNet121 model demonstrated superior performance in terms of accuracy when compared to the other techniques listed in the table. It achieved the highest accuracy among the reported models, making it a promising choice for image classification tasks. The Proposed DenseNet121 model achieved an accuracy of 97.01% for the BM Dataset. These results highlight the effectiveness of the integrated fine-tuned DenseNet121 model in the medical industry for classifying BM into seven classes. The accuracy achieved demonstrates the potential of this model for accurate and reliable diagnosis based on medical images. Table 3. Represents the comparison of the proposed study with State of Art.

## 5. Conclusions

This study presents a novel and practical framework, referred to as a classification diagnostic strategy, for classifying BM cells. The proposed method utilizes multiple TL models to detect and classify BM cells into seven categories: abnormal eosinophils, basophils, faggot cells, hairy cells, immature lymphocytes, smudge cells, and other cells. The primary objective of this approach is to assist medical professionals in accurately diagnosing various diseases associated with BM cells, such as leukemia. To complete this assignment, an analysis was conducted on five pre-trained models: EfficientNetB5, MobileNetV2, DenseNet121, ResNet50, and Xception. For this, all five pre-trained models were fine-tuned by adding a batch-normalization layer, a dropout layer and two dense layers. Among the five fine-tuned models, DenseNet121 demonstrated superior performance in terms of accuracy and loss. Then, this high-performance fine-tuned DenseNet121 model was optimized with four distinct optimizers and four different batch sizes for 25 epochs, with a learning rate of 0.0001. Further, the fine-tuned and optimized DenseNet121 model was integrated with an attention mechanism to improve its performance by allowing the model to focus on the most relevant features or regions of the image, which can be particularly beneficial in medical imaging where certain regions might have critical diagnostic information. The integrated DenseNet121 model with an attention mechanism demonstrated an accuracy rate of 97.01%. The acquisition of these measurements will allow analysts to create insights for advancing more effective models utilizing transfer learning models for categorizing BM.

## Figures and Tables

**Table 1:** List of hematological disease cell images included in the dataset.

Abbreviation	Meaning	Number of Images
ABE	Abnormal eosinophil	8
BAS	Basophil	441
FGC	Faggot cell	47
HAC	Hairy Cell	409
LYI	Immature lymphocyte	65
KSC	Smudge cell	42
OTH	Other cells	294

**Table 2:** Numbers of images in classes before and after data augmentation.

Class	Before Augmentation	After Augmentation
ABE	8	950
BAS	441	1055
FGC	47	1000
HAC	409	1030
LYI	65	990
KSC	42	995
OTH	294	1045

**Table 3:** SOTA on bone marrow cell classification.

Ref/Year	Technique	Dataset/Number of Images	Number of Classes	Accuracy
[12]/2021	ResNet50	Microscopic Lab Images/25,396	3	90%
[13]/2020	DC-GAN and ResNet	BCCD/12,447	4	91.68%
[14]/2021	VGG16 with InceptionV2	Microscopic Lab Images/20,670	2	90.00%
[26]/2021	Siamese Network for Few Shot	Hospital Dataset/-	2	89.66%
[28]/2021	ResNeXt	BM Dataset/170,000	21	94.8%
Proposed Model	Integrated fine-tuned DenseNet121 model withattention mechanism	BM Dataset/7065	7	97.01%

## Data Availability

The dataset used for this research work was taken from the Plant Pathology Dataset [28]. The datasets used to support the experimental outcomes of this study are available from the direct link in the dataset citations.
